# PrP^C^ expression and prion seeding activity in the alimentary tract and lymphoid tissue of deer

**DOI:** 10.1371/journal.pone.0183927

**Published:** 2017-09-07

**Authors:** Kristen A. Davenport, Clare E. Hoover, Jifeng Bian, Glenn C. Telling, Candace K. Mathiason, Edward A. Hoover

**Affiliations:** Prion Research Center, Microbiology, Immunology and Pathology Department, Colorado State University, Fort Collins, Colorado, United States of America; University of the Witwatersrand, SOUTH AFRICA

## Abstract

The agent responsible for prion diseases is a misfolded form of a normal protein (PrP^C^). The prion hypothesis stipulates that PrP^C^ must be present for the disease to manifest. Cervid populations across the world are infected with chronic wasting disease, a horizontally-transmissible prion disease that is likely spread via oral exposure to infectious prions (PrP^CWD^). Though PrP^CWD^ has been identified in many tissues, there has been little effort to characterize the overall PrP^C^ expression in cervids and its relationship to PrP^CWD^ accumulation. We used immunohistochemistry (IHC), western blot and enzyme-linked immunosorbent assay to describe PrP^C^ expression in naïve white-tailed deer. We used real-time, quaking-induced conversion (RT-QuIC) to detect prion seeding activity in CWD-infected deer. We assessed tissues comprising the alimentary tract, alimentary-associated lymphoid tissue and systemic lymphoid tissue from 5 naïve deer. PrP^C^ was expressed in all tissues, though expression was often very low compared to the level in the CNS. IHC identified specific cell types wherein PrP^C^ expression is very high. To compare the distribution of PrP^C^ to PrP^CWD^, we examined 5 deer with advanced CWD infection. Using RT-QuIC, we detected prion seeding activity in all 21 tissues. In 3 subclinical deer sacrificed 4 months post-inoculation, we detected PrP^CWD^ consistently in alimentary-associated lymphoid tissue, irregularly in alimentary tract tissues, and not at all in the brain. Contrary to our hypothesis that PrP^C^ levels dictate prion accumulation, PrP^C^ expression was higher in the lower gastrointestinal tissues than in the alimentary-associated lymphoid system and was higher in salivary glands than in the oropharyngeal lymphoid tissue. These data suggest that PrP^C^ expression is not the sole driver of prion accumulation and that alimentary tract tissues accumulate prions before centrifugal spread from the brain occurs.

## Introduction

Chronic wasting disease (CWD) is spreading among deer, elk and other cervids in North America, the Republic of Korea, and, recently, Norway [1–4]. The etiologic agent, a prion, results from the templated conversion from a normal protein, PrP^C^, to a primarily beta-sheet, misfolded form (PrP^CWD^). The prion hypothesis stipulates that the normal prion protein, PrP^C^, is necessary for the manifestation of PrP^CWD^ and the disease state [5]. As deer are likely exposed via the oral route to prions in the natural environment and because there is evidence that prions accumulate in the lymphoid tissue of cervids, we were interested in the expression of PrP^C^ in the alimentary tract and lymphoid system of the natural host (white-tailed deer, *Odocoileus virginianus*) [6–12]. The relationship between PrP^C^ expression and PrP^CWD^ accumulation in specific tissues has not been reported, though it is classically understood that the spread of prions involves centripetal spread from the peripheral nerves to the brain, followed by centrifugal spread to the rest of the body [13–16].

There is no literature describing the expression of PrP^C^ in cervids, the natural host of CWD. The expression of PrP^C^ has been described in cattle, the host of bovine spongiform encephalopathy, and sheep, the host of scrapie, and in common experimental models (hamsters and mice). In cattle, PrP^C^ has been described in the brain, lymphoid tissue, gastrointestinal nervous and mucosal tissues, thymus, kidney, heart, lung, liver, muscle and pancreas [17–20]. In sheep, PrP^C^ expression has been detected in the brain, intestine, lymphoid tissue, lung, heart, kidney, muscle, uterus, adrenal gland, salivary glands, stomachs and mammary glands [21, 22]. Finally, PrP^C^ has been identified in the muscle, alimentary tract, skin and respiratory epithelium of mice [23, 24] and in the CNS, lymphoid tissue, heart, liver, lung, kidney, stomach and intestine of hamsters [25–27]. We hypothesized that PrP^C^ would be widely distributed in white-tailed deer and that expression would be highest in lymphoid tissue, since lymphoid tissue plays a role in early CWD pathogenesis [6, 11, 12].

We were interested in the accumulation of PrP^CWD^ in alimentary tissues as well, since the oral route of exposure is likely responsible for horizontal transmission of CWD [28]. We hypothesized that tissues with the highest PrP^C^ expression would accumulate PrP^CWD^ earlier in disease, and that tissues with low PrP^C^ expression may never accumulate detectable PrP^CWD^ or only much later in disease. PrP^CWD^ has been detected in a number of deer tissues by a variety of methods, including western blot, immunohistochemistry (IHC) and enzyme-linked immunosorbent assay (ELISA). Tissues that have been identified as prion-positive in CWD-infected cervids include lymphoid tissues, brain, salivary glands, some parts of the intestinal tract, forestomachs, abomasum, pituitary, heart, adrenal gland, muscle, and fat [6, 9–11, 29–33].

We used ELISA and western blot to quantify PrP^C^ expression and IHC to describe to the distribution of PrP^C^ in 21 alimentary tissues and alimentary-associated lymphoid tissues of CWD-negative deer. We used real-time, quaking-induced conversion (RT-QuIC) to detect prion-seeding activity in the same tissues from orally-inoculated, CWD-positive deer either in the symptomatic stages of disease or at 4 months after oral inoculation with CWD (typically at least 12 months before we see clinical signs) [34]. We observed that PrP^C^ expression is widespread, and that seeding activity does not accumulate first in the tissues with the highest PrP^C^ expression. Importantly, we conclude that prion replication occurs in alimentary tissues before centrifugal spread of PrP^CWD^ from the brain. These results are an important step for understanding the pathogenesis of CWD and for understanding its facile horizontal transmission.

## Methods

### White-tailed deer husbandry, inoculation and necropsy

We maintained hand-raised white-tailed deer fawns (*Odocoileus virginianus*) indoors, in strict accordance with protocols specifically approved for this study by the Colorado State University Institutional Animal Care and Use Committee (protocols #13-4444A, #13-4610A, #13-4089A.) For euthanasia, we used Beauthanasia-D solution (phenytoin/pentobarbital) (IV) and for anesthesia, we used metedomidine and ketamine (IM via dart). The genotype of the deer at amino acid 96 was determined as previously described [35, 36]. Deer that were sacrificed in symptomatic stages of disease (1031, 1078, 1079, 1081, 1082) were inoculated *per os* with 0.01g (100μL of a 10% homogenate) of brain pooled from 6 CWD-positive, white-tailed deer. Specifically, we deposited the homogenate onto the back of the tongue of a sedated, but not yet anesthetized, deer and monitored the deer to ensure that the inoculum was swallowed and not aspirated. Deer sacrificed in pre-clinical stages of CWD (1171, 1201, 1205) were inoculated *per os* with 0.5g of the same brain homogenate using the same technique. CWD-negative deer were inoculated with 0.5g of CWD-negative brain homogenate (deer 1140, 1169, 1211) or were uninoculated (deer 952, 955). CWD-negative deer were housed in separate suites in the same indoor facility as the CWD-inoculated deer [37].

### Tissue collection and processing

We collected each tissue with new, prion-free, disposable instruments. For GI tissues, we removed a cross-section of tissue and let loose ingesta fall from the section. We then froze half of each tissue at -80°C and fixed the other half in periodate-lysate-paraformaldehyde (PLP) for 4 days. After fixing, we stored the tissues in sterile PBS until trimming, followed by 70% ethanol for long-term storage. We trimmed tissues into cassettes and embedded them in paraffin blocks using routine histologic techniques.

We homogenized frozen full-thickness (GI) or cross-sectioned (lymph node) tissue for western blots, RT-QuIC, and ELISA using the following protocol: we trimmed approximately 200mg of tissue on ice and added it to ice-cold 1X PBS with protease inhibitors (Roche Complete Mini protease inhibitor tablets; one tablet/10mL PBS) to create a 20% w/v homogenate. We homogenized the tissues (Bead Ruptor 24 Bead Mill Homogenizer, Omni International) for 30 seconds, followed by a 10 second pause, then another 30 second homogenization. We chilled the homogenates on ice for 5 minutes, then repeated the homogenization and chill protocol twice more. We diluted each sample to 1% for the bicinchoninic acid (BCA) assay (Pierce BCA Protein Assay Kit, Thermo Scientific), and froze the remaining 20% homogenates at -80°C.

We performed BCA assays according to the manufacturer’s instructions, with each sample and standard tested in triplicate. We recorded optical density with an Opsys MR microplate reader (Dynex technologies).

### Western blot

We thawed frozen 20% tissue homogenates on ice and added 500μg of total protein to 2% N-lauroylsarcosine sodium salt in PBS for a final volume of 150μL. We thawed brain homogenates on ice and added 5.0ug of total protein to 2% sarkosyl in PBS for a final volume of 150μL. We added 25μL of 6X Laemmli sample buffer to each sample, then boiled for 5 minutes. Next, we loaded 20μL of each sample preparation (~57μg total protein for tissues and 0.57μg total protein for brain) to each well of an 18-well precast gel (12% Criterion™ XT Bis-Tris protein gels, Bio-Rad) and electrophoresed the gels for 90 minutes at 150V. We transferred the proteins to a PVDF membrane for 1 hour at 80V on ice (Criterion™ Blotter, Biorad), then blocked the membranes with 5% non-fat dry milk in TBST for 20 minutes at room temperature, then incubated the membrane with 0.2μg/mL antibody Bar224 (Cayman Chemicals) overnight at 4°C (monoclonal antibody raised against ovine PrP^C^, amino acids 141–151). We followed the primary antibody with a horseradish-peroxidase-labeled goat anti-mouse IgG for one hour at room temperature and developed the western blots with Pierce ECL substrate (ThermoFisher Scientific) for 5 minutes. We captured images with the GE ImageQuant LS 400 imager; specifically, we exposed the membranes for 70 seconds and analyzed the western blots whose bands were uninterrupted by bubbles or other artifacts.

We used Image Studio Lite v4.0 (Li-Cor Biosciences) for densitometry. First, we drew a rectangle around the first lane and copied it onto each lane, then used the software’s background subtraction tool, which subtracted the median background from the right and left of each rectangle for 3 border-widths. Next, we calculated the intensity of each lane relative to the brain sample that was included in every experiment. We plotted a frequency histogram of all lane intensities from every experiment and divided the data evenly into 4 quadrants. We used the borders of these quadrants as cutoffs for our final classification (very low, low, medium and high).

### Histology and immunohistochemistry

BAR224: We mounted 5μm sections of paraffin-embedded tissue on positively-charged glass slides. We heated the slides at 65°C, then removed paraffin with xylene and rehydraded the tissues in graded alcohols (100%, 95%, 70%) and water. We treated the tissues with 88% formic acid for 5 minutes to expose epitopes. To quench peroxidases, we incubated the tissues with 3% hydrogen peroxide in methanol. We blocked tissues with 5% non-fat dry milk (in TNT [0.1M Tris-HCl, 150mM NaCl, 0.1% Tween]). We incubated tissues overnight at 4°C in a bath of 2μg/mL anti-PrP^C^ antibody Bar224 (Cayman Chemicals) or mouse IgG_2A_ as a negative control (RD Systems). We incubated the tissues with secondary anti-mouse IgG conjugated to horseradish peroxidase (Envision+™, Dako) at room temperature. Finally, we detected the immunoreactivity with AEC chromagen (Dako). We counterstained with Mayer’s hematoxylin (Dako), followed by 0.1% sodium bicarbonate bluing reagent, then added coverslips with aqueous mounting media (Dako) and allowed to dry completely.

12B2: We mounted 5μm sections of paraffin-embedded tissue on positively-charged glass slides. We heated the slides at 65°C, then removed paraffin with xylene and rehydraded the tissues in graded alcohols (100%, 95%, 70%) and water. We used heat-induced epitope retrieval with citrate buffer (10mM sodium citrate, pH 6.0) to expose epitopes. To quench peroxidases, we incubated the tissues with 3% hydrogen peroxide in methanol. We blocked tissues with TNB buffer (PerkinElmer). We incubated tissues overnight at 4°C with 17μg/mL anti-PrP^C^ antibody 12B2 (Wageningen Bioveterinary Research) or mouse IgG_1-k_ as a negative control (VWR-Biolegend). We incubated the tissues with secondary anti-mouse IgG conjugated to horseradish peroxidase (Envision+™, Dako) at room temperature. Finally, we detected the immunoreactivity with AEC chromagen (Dako). We counterstained with Mayer’s hematoxylin (Dako), followed by 0.1% sodium bicarbonate bluing reagent, then added coverslips with aqueous mounting media (Dako) and allowed to dry completely.

For hematoxylin and eosin staining, we used the same deparaffinization protocol. We stained the tissues with Mayer’s hematoxylin, then eosin (NovaUltra™ H&E Stain Kit, IHC World). We cleared the slides in xylene, then added coverslips with xylene-based mounting media and allowed to dry. We visualized staining with light microscopy.

### Enzyme-linked immunosorbent assay

We coated 96-well plates (Maxisorp plates, Nunc) with 20μg/mL capture antibody D18 [38] (Telling laboratory, Colorado State University Prion Research Center) in carbonate/bicarbonate buffer, sealed and stored for 1–4 days at 4°C. We blocked the plates with 3% bovine serum albumin (BSA) in PBS at 37°C. We thawed tissue homogenates on ice and added 250–2000μg/mL total protein to 0.23% BSA, 0.035% Triton X100 and PBS. We added 100μL of each sample to three wells and incubated overnight at 4°C. We added PRC5 (0.27μg/mL, Prion Research Center, Colorado State University [39]) in 1% BSA in PBS to the plates at 37°C, then washed and added anti-mouse IgG2s-HRP conjugate (Alpha Diagnostic International Inc) at a 1:5000 dilution at 37°C. We developed the plates with ABTS peroxidase substrate (Thermo Fisher Scientific), then stopped the reaction. We read the absorbance at 405nm on an ELx808 Ultra Microplate Reader (Bio-Tek Instruments, Inc.). In addition to the samples of interest, we included blank wells and standard wells. The blanks were missing only the tissue homogenates and the standards included recombinant white-tailed deer rPrP instead of tissue homogenate. We used concentrations of 0.1-1250ng/mL rPrP for the standard curve.

We assessed each sample for its linearity. Samples that did not exhibit a linear response to dilution were repeated at higher total protein concentrations. Samples that did not cross the threshold (3 standard deviations above the mean absorbance of the blanks) were also repeated with higher total protein concentrations. If samples exhibited a linear response of absorbance to dilution and had at least two dilutions above the threshold, we proceeded with analysis. We were able to proceed with analysis of all tissues except the following: 1/3 rumen samples (below threshold), 1/3 spleen samples (nonlinear dose response), 2/3 prescapular lymph node samples (nonlinear dose response), 1/3 omasum samples (below threshold), and 1/3 ileocecocolic LN (nonlinear dose response). We computed the linear regression for the standard curves (within the linear range) and extrapolated the ng/mL PrP^c^ for each sample (choosing the dilution in the center of the linear range). We divided the ng/mL PrP^c^ by the μg/mL total protein added to the ELISA to calculate a ratio of PrP^c^/total protein for each sample. We plotted a frequency histogram of the ng PrP^C^/mg total protein for every replicate and divided the data into quartiles. We assigned a score of 4 to replicates that fell within the highest quartile, 3 for the 50–75% quartile, 2 for the 25–50% quartile and 1 for the lowest quartile. We averaged the scores of each replicate (n = 3) and each deer (n = 3). A score of 1 represents less than 1.36 ng PrP^C^/mg total protein, a score of 2 represents 1.37–2.51ng PrP^C^/mg total protein, a score of 3 represents 2.52–4.77ng PrP^C^/mg total protein and a score of 4 represents greater than 4.78ng PrP^C^/mg total protein. We compared sample types or groups of samples with a one-way ANOVA, followed by Tukey’s multiple comparison post-test.

### Expression and purification of recombinant PrP

We expressed full-length white-tailed deer (WTD) PrP^c^ for standard curves in ELISA as previously reported [40] and truncated Syrian hamster (SH) PrP^c^ for RT-QuIC as previously reported [41]. Briefly, the cDNA sequence for the WTD *PRNP* gene was cloned into the pet100D expression system (Life Technologies). The plasmid for expression of amino acids 90–231 of SH PrP^c^ was kindly provided by Dr. Byron Caughey. We stored the plasmids in *E*. *coli* BL21 Star cells (Life Technologies). To express PrP^c^, we added BL21 cells from frozen glycerol stocks to 5mL LB media, grew the cultures overnight, then added the bacteria to 1L LB media with auto-induction reagents (final concentration: 0.5M (NH_4_)_2_SO_4_, 1M KH_2_PO_4_, 1M Na_2_HPO_4_, 0.5% glycerol, 0.05% glucose, 0.2% α-lactose and .001M MgSO_4_.) We harvested bacteria when the OD_600_ reached approximately 3.0 for WTD and 1.7 for SH PrP. We lysed the cells and purified inclusion bodies according to the manufacturer’s protocol with BugBuster™ and Lysonase™ (EMD-Millipore).

To purify recombinant PrP (rPrP), we solubilized the inclusion bodies in 8M guanidine hydrochloride (GdnHCl) and 100mM Na_2_HPO_4_ at room temperature, overnight, in an end-over-end rotator. We mixed the denatured rPrP slurry with Superflow™ nickel resin (Qiagen) and refolded the rPrP on the column and eluted as previously reported [40].

### Real-time quaking-induced conversion (RT-QuIC) with NaPTA precipitation

We thawed aliquots of frozen tissue homogenates on ice and diluted the homogenate to 0.1% in 0.1% SDS/PBS. We added sodium phosphotungstic acid (NaPTA) (final concentration 0.33% NaPTA, 0.28% MgCl_2_) to 100μL 0.1% tissue homogenate. The solution was shaken at 1400rpm for 1 hour at 37°C, then the precipitated proteins were pelleted at 14,000g for 30 minutes. We resuspended NaPTA pellets in 10μL 0.1% SDS/PBS and added 2μL to each well of the RT-QuIC plate. We did not subject brain samples to NaPTA precipitation; we diluted 10% brain homogenates in 0.1% SDS/PBS and tested the 10^−3^ and 10^−4^ dilutions.

We performed RT-QuIC experiments in black, optical-bottom 96-well plates (Nunc). Each RT-QuIC reaction was comprised of 0.1mg/mL SH rPrP, 320mM NaCl, 1.0mM EDTA, 20mM NaH_2_PO_4_ and 10μM thioflavin T. An RT-QuIC experiment consisted of 250 cycles (62.5 hours) of shaking (1 minute, double orbital shaking at 700rpm) and rest (1 minute) at 40°C. Microplate readers (Fluostar, BMG) recorded the fluorescence (450nm excitation and 480nm emission) every 15 minutes using a gain of 1700.

We included matched CWD-negative control tissues on every plate and samples were run in quadruplicate twice (8 total replicates). We calculated a threshold at 5 standard deviations above baseline fluorescence to determine which replicates were positive. We used Omega software (BMG Labtech) to determine the lag phase—the time at which the fluorescence in a given well exceeded the threshold. If the fluorescence for a sample never crossed the threshold during the experiment, we estimated a value of 70 hours. We analyzed the lag phase data using non-parametric, rank-based tests (Wilcoxon-Mann-Whitney (WMW) and Wilcoxon signed-rank tests). If all values for one sample were equal, WMW tests are invalid, so we used Wilcoxon signed-rank tests instead. We chose these tests for several reasons: 1) our data was often non-normal (right skewed due to replicate wells that don’t cross the threshold in the course of the experiment); 2) our n was relatively small (8); and 3) we chose values to substitute for the replicate wells that did not cross the threshold. The choice of that value would affect the mean (and the results of any statistical tests that relies on means, like t-tests), but will not affect rank-based statistical analysis, since those values will have the longest lag phase regardless of which value we choose. We described a tissue as positive when it was statistically different from the matched tissue from a CWD-negative deer (p<0.05).

## Results

### PrP^C^ expression is widespread, but low, in many tissues of the alimentary tract and alimentary-associated lymphoid tissue

**Fig 1:**
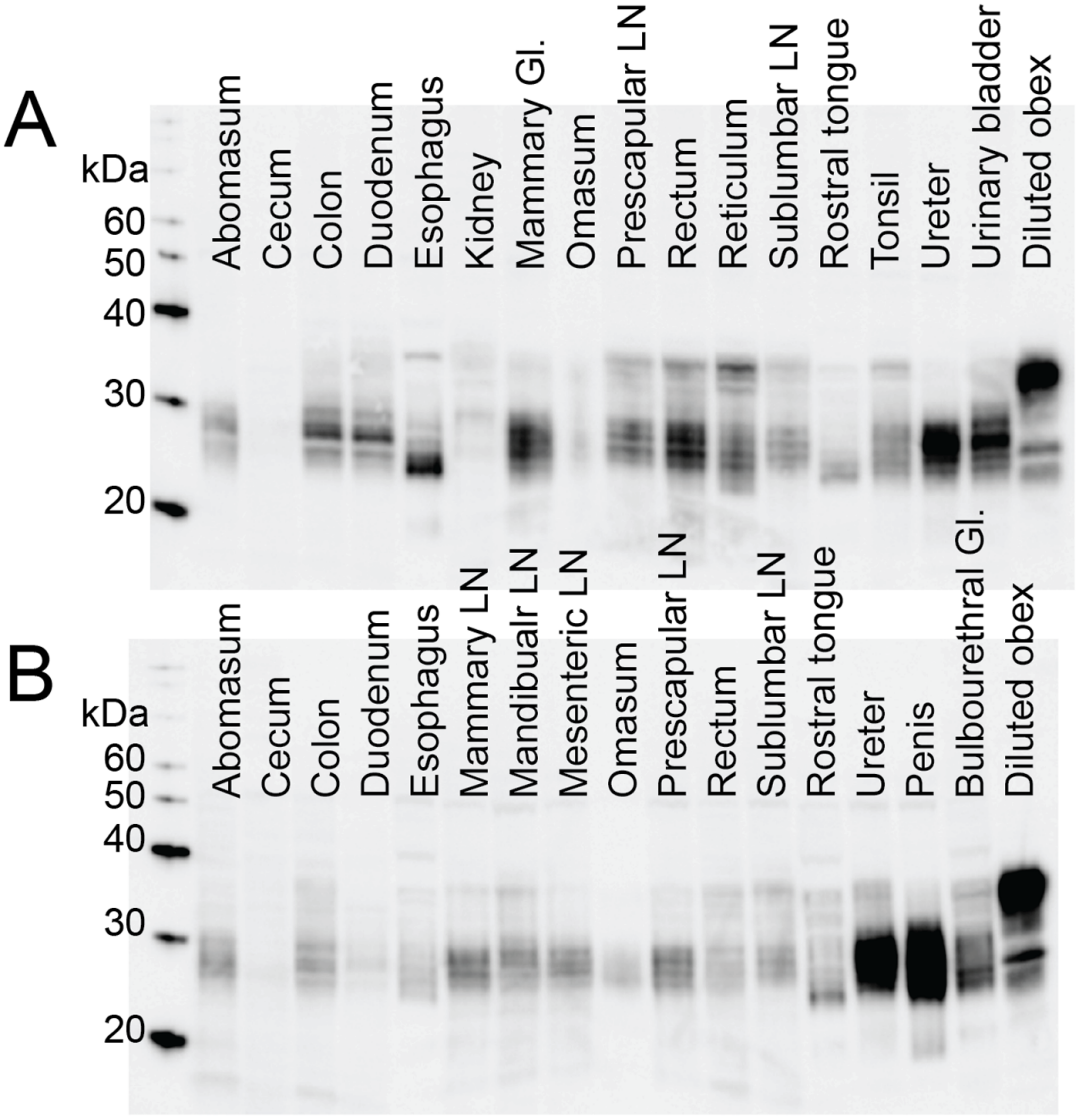
Representative western blots. We performed western blots for PrP^C^ expression on tissue homogenates from 5 naïve deer. We used a diluted obex homogenate (1/100 of total protein added for other samples) to normalize each western blot (lane 18) and performed densitometry on each lane. A) Representative western blot from deer 952. B) Representative western blot from deer 955. Expression of PrP^C^ was variable among tissues and, to some degree, between deer. However, we were able to detect PrP^C^ in every tissue in at least one of the deer. Banding patterns were variable among tissues, which may reflect differences in cleavage products and/or in sample degradation.

**Fig 2:**
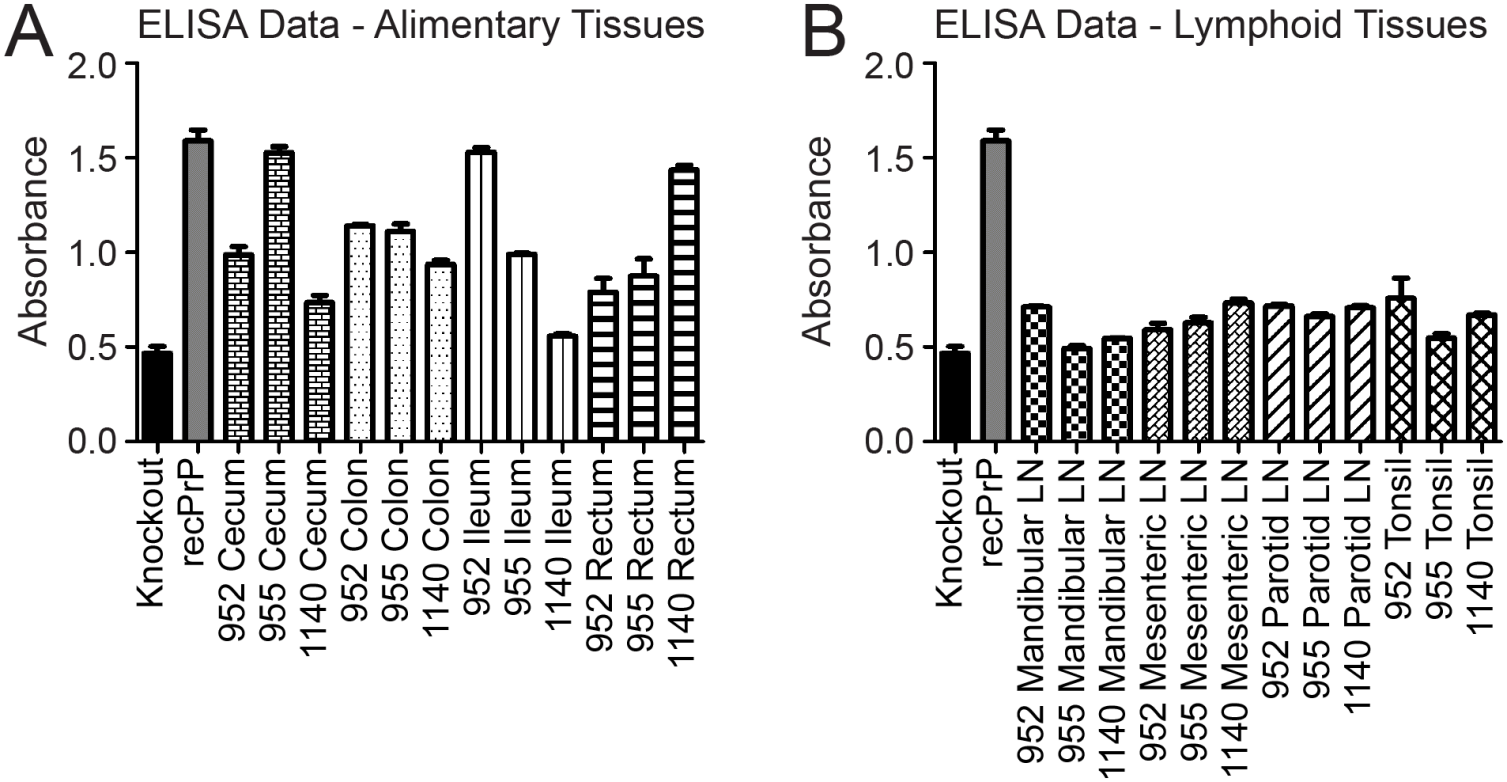
Representative ELISA results. We performed sandwich ELISA assays for PrP^C^ expression on tissue homogenates from 3 naïve deer. We used PrP^0/0^ mouse tissues as negative controls and 5 dilutions of recombinant white-tailed deer PrP^C^ (recPrP) to generate a standard curve, one dilution of which is shown here. Bars represent the mean absorbance of three technical replicates and error bars represent standard error of the mean. A. We compared several alimentary tissues from all three deer. B. We compared several alimentary-associated lymphoid tissues from all three deer. Replicate samples from individual deer were very consistent (error bars), but there was some variability between deer for a given tissue (note differences in bars of the same pattern). Overall, expression was higher in the alimentary tissues than in the alimentary-associated lymphoid tissues.

**Fig 3:**
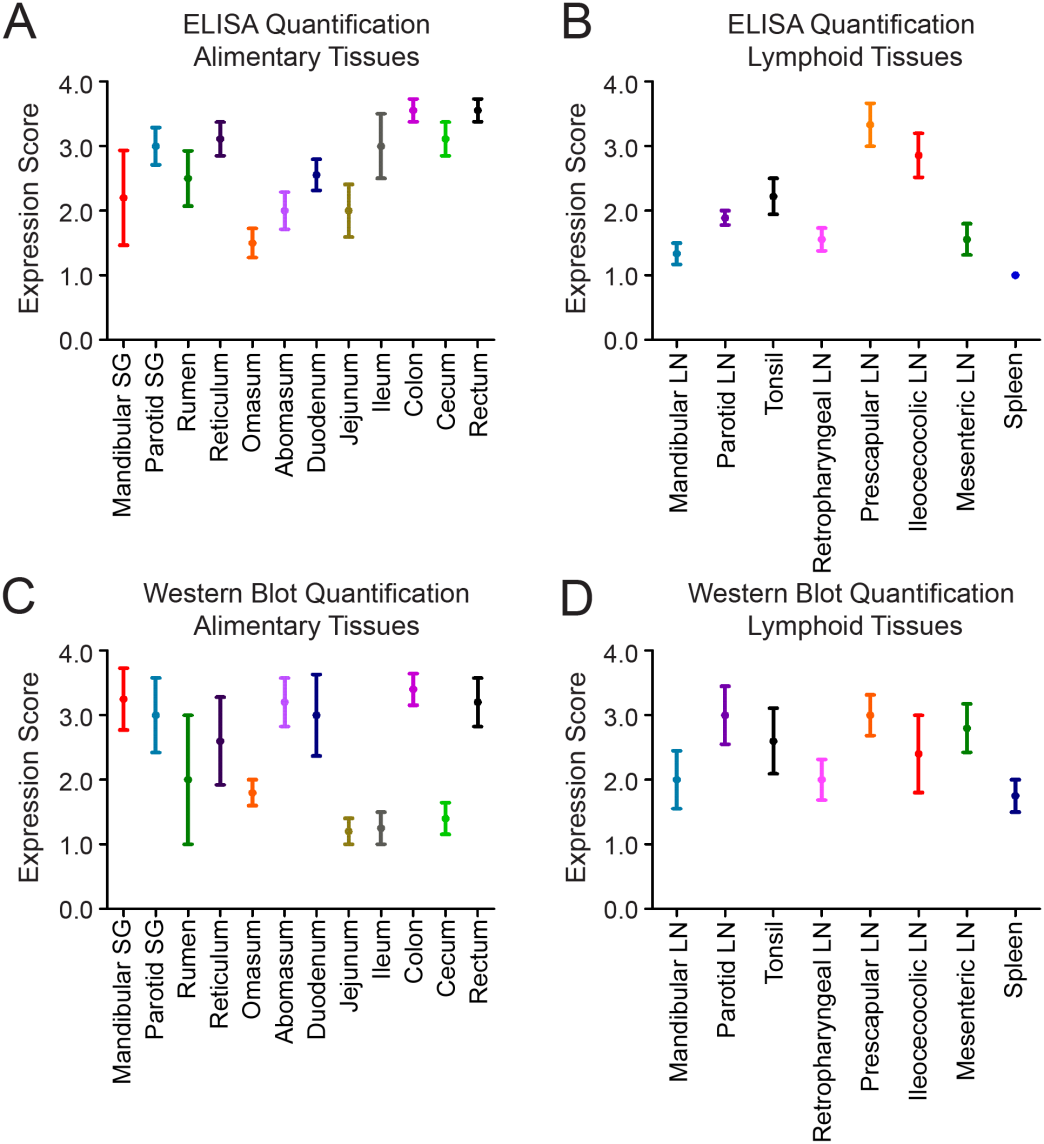
Quantification of PrP^C^ expression. A-B. We converted ELISA absorbance values to expression scores using the approach detailed in the methods section. Briefly, we divided every replicate’s absorbance into quartiles and assigned the top quartile a score of 4 and the lowest quartile a score of 1. For every tissue, the mean score for 3 replicates for each of three deer is plotted with the standard error of the mean. We divided the tissues we tested into alimentary (Fig 3A) and lymphoid tissues (Fig 3B). C-D. We converted western blot densitometry data to expression scores using the technique described in the methods. Briefly, we normalized each lane to the diluted obex on the same blot, then divided all normalized densitometry data into quartiles. The top quartile received a score of 4 and the lowest received a score of 1. We plotted the mean score for 5 deer with the standard error of the mean. We divided the tissues into alimentary (including glandular) (Fig 3C) and lymphoid tissues (Fig 3D). The error bars indicate that the alimentary tissues were more variable than the lymphoid tissues and that the western blot results were more variable than the ELISA results.

We used western blots and enzyme-linked immunosorbent assays (ELISA) to detect PrP^C^ in frozen tissue samples from captive, CWD-naïve, white-tailed deer. We examined 21 tissues of the alimentary tract, alimentary-associated lymphoid tissues, and peripheral lymphoid tissues. We classified PrP^C^ expression in each tissue into quartiles and assigned expression scores based on the quartile where the tissue fell. We detected PrP^C^ expression in all tissues by western blot, although expression was often near the limit of detection (representative western blots in Fig 1). We were able to detect PrP^C^ expression by ELISA in nearly all tissues (select raw ELISA data in Fig 2) Overall, the assays detected similar expression in a given tissue. When expression levels varied between assays, the ELISA detected higher PrP^C^ expression than did western blot (ileum and cecum), with the exception of the mesenteric LN (Fig 3). For the statistical analysis, we used the ELISA data, since it is inherently the more quantitative assay [23].

**Fig 4:**
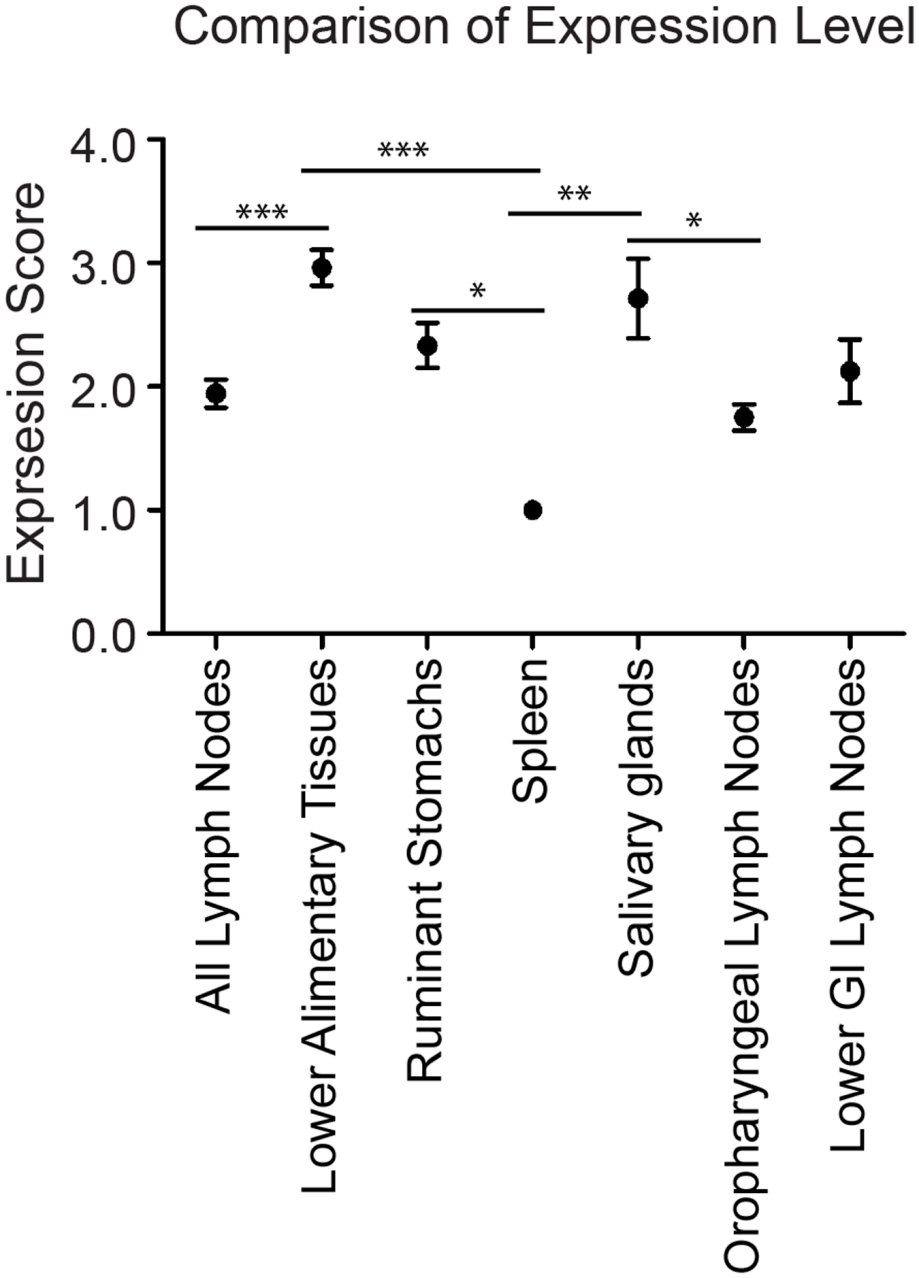
Comparison of PrP^C^ expression among tissue types. We used one-way ANOVA and Tukey’s Multiple Comparison post-test to analyze differences between PrP^C^ expression levels in types of tissue (based on ELISA results.) * indicates p<0.05, ** p<0.01, *** p<0.0001. *Lower alimentary tissues* include: duodenum, jejunum, ileum, cecum, colon and rectum. *Ruminant stomachs* include: rumen, reticulum, omasum and abomasum. *Oropharyngeal lymph nodes* include: tonsil, retropharyngeal lymph node, mandibular lymph node and parotid lymph node. *Lower GI lymph nodes* includes ileocecocolic lymph node and mesenteric lymph node. As indicated by the asterisks, there are statistically significant differences between the following groups: lower alimentary tissues > all lymph nodes; lower alimentary tissues > spleen; ruminant stomachs > spleen; salivary glands > spleen; salivary glands > oropharyngeal lymph nodes. We plotted means and standard error of the mean.

We were interested in a comparison of alimentary-associated lymphoid tissue (which is assumed to play a major role in CWD pathogenesis) and tissues of the alimentary tract. Tissues of the lower alimentary tract (small and large intestines and rectum) had higher PrP^C^ expression than did the group comprised of all the alimentary-associated lymphoid tissue (ANOVA, Tukey post-test, p<0.05) (Fig 4) Earliest CWD prion replication was detected in oropharyngeal lymphoid tissue, so we compared PrP^C^ expression in these tissues (mandibular LN, parotid LN, tonsil, retropharyngeal LN) to the salivary glands, which are also proximate to the oral cavity and are likely involved in the contamination of saliva with prions [12, 30, 41, 42]. The salivary glands had higher PrP^C^ expression than did the oropharyngeal lymph nodes (ANOVA, Tukey post-test, p<0.05) (Fig 4). However, there was no difference in the PrP^C^ expression of the oropharyngeal lymph nodes and the lymph nodes of the lower GI (ANOVA, Tukey post-test, p>0.05) (Fig 4). Finally, we were interested in the PrP^C^ expression level in the spleen, which had lower expression than the lower alimentary tract tissues, the salivary glands and the ruminant stomachs (ANOVA, Tukey post-test, p<0.05). The spleen also had (statistically insignificantly) lower PrP^C^ expression than all lymph nodes combined (Fig 4).

### PrP^C^ is expressed in the stratified squamous epithelium of alimentary tissues, in addition to the nervous tissue components

**Fig 5:**
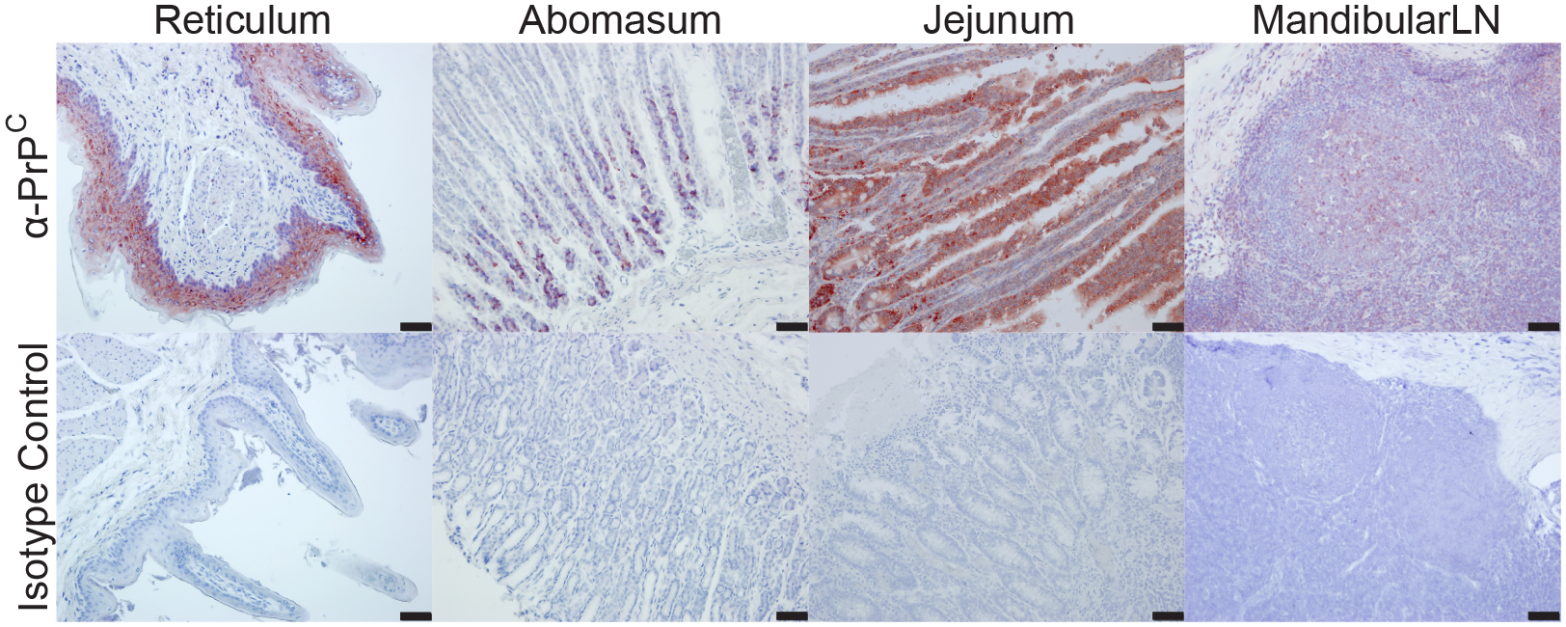
Representative IHC results. We have displayed 4 tissues that represent the most common PrP^C^ staining patterns from our IHC experiments. All four tissues are stained with an anti-PrP antibody or an isotype control antibody. Images are 200X; scale bar represents 50μm. Descriptions of the staining patterns are in Table 1.

To assess the distribution of PrP^C^ in CWD-negative deer tissues, we performed IHC with anti-PrP^C^ antibodies to PrP^C^ epitopes 97–115 and 141–151. We observed PrP^C^ immunoreactivity in mucosal stratum spinosum and stratum granulosum epithelial cells of the rumen, reticulum, and omasum forestomachs (Fig 5, Table 1, S1 Table). In the abomasum, we detected PrP^C^ immunoreactivity in individual cells in the gastric pits with a phenotype consistent with peptic chief cells (Fig 5, Table 1, S1 Table). We observed variable immunoreactivity in the crypt and villar enterocytes of the small intestinal mucosa (Fig 5, Table 1, S1 Table). In the cecum and colon, we observed the majority of PrP^C^ in histiocytic cells residing in the mucosal lamina propria. The greatest and most consistent PrP^C^ immunoreactivity was in myenteric plexi throughout the entire alimentary tract and in germinal centers of gastrointestinal-associated lymphoid tissue. For every tissue and each anti-PrP^C^ antibody, we treated a matching slide with an isotype control antibody, which consistently failed to result in staining.

**Table 1:** Summary of IHC results. Results from IHC are summarized below. Gray indicates staining in the structure (row) for each tissue (column). (Ru = rumen, Ret = reticulum, Om = omasum, Ab = abomasum, Du = duodenum, Je = jejunum, Il = ileum, Ce = cecum, Co = colon, Re = rectum, Pa SG = parotid SG, T = tonsil, R LN = retropharyngeal LN, Ma LN = mandibular LN, Par LN = parotid LN, Me LN = mesenteric LN, Ile LN = ileocecocolic LN, Pre LN = prescapular LN, S = spleen.

**Alimentary tissues** *Staining present in*:	Ru	Ret	Om	Ab	Du	Je	Il	Ce	Co	Re	Pa SG
Mucosal epithelial cells											
…stratum spinosum and granulosum											
… gastric pits											
…mucosal enterocytes											
Mucosal histiocytic cells											
Myenteric plexi											
Acinar cells											
**Lymphoid tissue** *Staining present in*:	T	RLN	Ma LN	Par LN	Me LN	Ile LN	Pre LN	S			
Germinal centers											
Mantle Zone											
Paracortex											
Sinus histiocytic cells											

### PrP^C^ is expressed in germinal centers of lymphoid tissue

We observed diffuse immunoreactivity in germinal centers of lymphoid follicles that occasionally extended to the mantle zone (Fig 5, Table 1, S1 Table). In multifocal regions of the paracortex, cells displayed cytoplasmic staining. In some lymph nodes, a mild to moderate number of histiocytic-like cells in the subcapsular and medullary sinuses had marked cytoplasmic staining, often in cells surrounding blood vessels (Table 1, S1 Table).

### Prion seeding activity is widespread in alimentary tissues and alimentary-associated lymphoid tissue

Our observation that PrP^C^ expression was widespread, albeit low, prompted us to investigate the distribution of PrP^CWD^ seeding activity in these same tissues in CWD-infected deer. We examined all 21 tissues (plus the obex region of the brain stem) by real-time quaking-induced conversion (RT-QuIC) with NaPTA precipitation to eliminate spontaneous conversion. In deer sacrificed after clinical signs developed, every tissue we tested was positive by RT-QuIC (statistically different from the paired negative control) in all 5 deer (Table 2).

**Table 2:** Distribution of prion seeding activity in CWD-infected white-tailed deer. We performed real-time, quaking-induced conversion with NaPTA pre-treatment on homogenates of the same tissues in CWD-inoculated white-tailed deer. We included 5 deer that were euthanized in symptomatic stages of disease and 3 deer that were euthanized 4 months after oral inoculation with CWD-positive brain tissue (4mpi, no signs of CWD). We compared inoculated deer to CWD-negative deer; for a tissue to be designated as positive, it must have been significantly different from the negative control tissue tested in the same experiment (Wilcoxon-Mann-Whitney or Wilcoxon Signed Rank tests, p<0.05). We have listed the number of positive deer/ number of total deer in this table. The darker shades indicate tissues where a greater proportion of deer were positive. Clear wells had no positive results. We classified tissues into lymphoid or alimentary categories. We tested the obex region of the brainstem to assess whether the deer had reached the neuroinvasion stage.

**Prion seeding activity in mucosal tissues**		
**Tissue**	Terminal Deer	4mpi Deer
**Mandibular SG**	**5/5**	**0/3**
**Parotid SG**	**5/5**	**2/3**
**Rumen**	**5/5**	**0/3**
**Reticulum**	**5/5**	**0/3**
**Omasum**	**5/5**	**1/3**
**Abomasum**	**5/5**	**1/3**
**Duodenum**	**5/5**	**0/3**
**Jejunum**	**5/5**	**0/3**
**Ileum**	**5/5**	**3/3**
**Colon**	**5/5**	**1/3**
**Cecum**	**5/5**	**0/3**
**Rectum**	**5/5**	**0/3**
**Prion seeding activity in lymphoid tissues**		
**Tissue**	**Terminal Deer**	**4mpi Deer**
**Mandibular LN**	**5/5**	**3/3**
**Parotid LN**	**5/5**	**3/3**
**Tonsil**	**5/5**	**3/3**
**Retropharyngeal LN**	**5/5**	**3/3**
**Prescapular LN**	**5/5**	**3/3**
**Ileocecolic LN**	**5/5**	**3/3**
**Mesenteric LN**	**5/5**	**2/3**
**Spleen**	**5/5**	**3/3**
**Prion seeding activity in the CNS**		
**Obex**	**5/5**	**0/3**

Other work in our laboratory demonstrated that by 4 months post-oral inoculation with CWD, deer have substantial prion seeding activity in many lymphoid tissues, but not in brain [12]. We were curious to test the alimentary tract tissues from these subclinical deer to learn which tissues are involved before prion amplification is detectable in the brain. Indeed, some of the alimentary tissues were positive (statistically different from the CWD-negative deer) in some deer 4 months post inoculation (Table 2). Involvement of the ileum was not surprising, given the extent of lymphoid tissue and Peyer’s patches in the ileum, but the omasum, abomasum, colon and salivary glands have little appreciable lymphoid tissue and demonstrated significant prion seeding activity.

## Discussion

The prion hypothesis postulates that the normal prion protein, PrP^C^, must be present in an individual tissue for the disease to manifest, since it is absolutely necessary for the generation of pathogenic PrP^CWD^ prions [43, 44]. Though the presence of PrP^CWD^ has been demonstrated in many tissues in CWD-infected cervids, there has been no comprehensive description of PrP^C^ expression in the natural host [6, 9–11, 29–33]. Several questions about the pathogenesis of CWD and the mechanism by which prions spread through the host remain unanswered: (1) is PrP^C^ substrate available for conversion to PrP^CWD^ in the tissues of deer where prions are detected? And (2) do prions accumulate earliest in the tissues with the highest PrP^C^ expression? We began these studies with the hypothesis that prions accumulate early in disease in tissues where PrP^C^ expression is highest. We found that PrP^C^ expression was widespread and is not highest in the tissues where prions first accumulate. Specifically, PrP^C^ expression was higher in the lower GI tissues than in the alimentary-associated lymphoid system and higher in salivary glands than in the oropharyngeal lymphoid tissue. However, the variable seeding activity in salivary glands and lower GI tissues of subclinical deer (compared to consistent seeding activity in the lymphoid tissues) suggests that seeding activity accumulates in the lymphoid tissues before lower GI tissues or salivary glands (Table 2).

We were interested in the tissues of the alimentary tract because, in nature, cervids are most likely exposed to CWD prions via the oral route. We found that PrP^C^ expression was widespread in the alimentary tract, albeit at variable levels (Fig 3A and 3C). We describe PrP^C^ expression in the myenteric plexi of the GI tract (where PrP^CWD^ has been demonstrated [9, 10, 29]) (Table 1). PrP^C^ was also present in the mucosal stratum spinosum and granulosm layers and in follicle-associated epithelium of Peyer’s patches (Table 1). The follicle-associated epithelium contains M cells, whose role in prion uptake has been demonstrated in mice with scrapie [45–47]. While the function of PrP^C^ in alimentary epithelia is unclear, Morel, et al. demonstrated the expression of PrP^C^ by enterocytes and a role for PrP^C^ in the maintenance of cell-cell junctions and cellular division [48, 49]. There is evidence that enterocytes play a role in sampling the intestinal lumen, which suggests that they may play a role in the passage of PrP^Sc^ into the sub-mucosal lymphatics [50, 51]. It is interesting to consider whether epithelial PrP^C^ plays a role in prion uptake, serves as a substrate for conversion to PrP^Sc^, or some other role. An important future experiment involves a sensitive IHC assay for the detection of PrP^Sc^ in the alimentary tissues whose PrP^C^ expression we investigated here. The correlation of PrP^Sc^ and PrP^C^ in these tissues may shed some light on the involvement of alimentary PrP^C^ in the disease process.

We hypothesized that lymph nodes would have high PrP^C^ expression compared to non-lymphoid, alimentary tissues, since they play a role in early CWD pathogenesis and are prion-positive by many methods in symptomatic disease [6–12]. However, expression of PrP^C^ in lymph nodes was fairly low overall (Fig 4), but was more consistent among deer than were PrP^C^ expression levels in the other tissues (error bars in Fig 3). Because PrP^C^ expression is highest in lymphoid follicles, it is possible that inconsistency in the number of follicles in the homogenized tissues could cause expression to seem appear low. However, we saw very consistent results among deer, which suggests that the follicle content was fairly consistent. These results suggests that the importance of lymph nodes early (and throughout) CWD progression may not be because they have the highest pool of available PrP^C^ substrate, but rather because of their specific immunosurveillance role in the alimentary tract, transporting and presenting antigens. This hypothesis is supported by germinal center staining in lymphoid follicles, consistent with B cells and follicular dendritic cells, and mantle zone staining, consistent with resting B cells [6]. B cells have been implicated in the uptake and trafficking of prions and in the transfer of prions to follicular dendritic cells [52]. B cells from CWD(+) deer are infectious to naïve deer [37], supporting the hypothesis that lymphoid cells play a specific role in CWD pathogenesis that does not rely on high PrP^C^ expression. In sheep, PrP^C^ is expressed in the spleen [22] and the spleen is a site of prion accumulation [53–55] and a source of infectious prions [56]. The spleen has been identified as an early, but not required, site of prion replication in mouse-adapted scrapie [13, 16, 57, 58], but similar to Syrian hamsters, PrP^C^ expression in the deer spleen is relatively low compared to other tissues [27] (Figs 3 and 4).

A potential caveat of the present study is the duration of storage for the CWD-negative deer tissues. All tissue samples used were either conventionally frozen at -80°C or paraformaldehyde-fixed, thereby preventing assays that require preserved mRNA (RT-PCR) or fresh, unfixed tissues (flow cytometry). Because western blots are inherently semi-quantitative, we performed our statistical analyses with ELISA results. It is also plausible that tissue sections from different deer vary in their relative connective tissue, fat, stroma, etc. content. Our IHC data indicate that PrP^C^ expression is specific to particular cell types, so a difference in the proportion of that cell type in the tissue homogenate could cause the expression level to vary by western blot or ELISA. We do not suspect falsely elevated expression levels in deer tissue due to components other than PrP^C^ because the knockout mouse tissues used as negative controls in western blot and ELISA produced no signal above background (Fig 2). We would also expect the tissue milieu to be similar among individual deer.

Several authors have described a centrifugal spread of prions, in which amplification occurs in the brain, and prions spread through the body along peripheral nerves [1, 14, 15, 59]. Prions have been hypothesized to reach the brain via peripheral nerves, including those in the GI tract, [13, 14, 60], and it is clear that prion inoculum can cross the mucosal barrier of the alimentary tract very soon after exposure [61]. We tested alimentary and alimentary-associated lymphoid tissues of deer in the symptomatic stages of CWD and at 4mpi for the presence of prion seeding activity by RT-QuIC. In symptomatic animals, all examined tissues were positive, including the obex region of the brainstem (Table 2). However, at 4mpi, prions were widespread in alimentary-associated lymphoid tissues, and were detectable in a few alimentary tissues (Table 2). At 4mpi, the obex was still negative, which suggests that the positive alimentary tissues were not positive due to the centrifugal spread of prions from the CNS to the myenteric plexi of the peripheral nervous system. We hypothesize that prions replicate in GI tissues, and demonstrate that prions found in the GI tract are not all derived from the CNS. The lymphoid tissue of 4mpi deer had seeding activity in every deer, but the seeding activity in alimentary tissues was variable among deer. This difference suggests that 4mpi falls in the midst of the progression from lymphoid involvement to involvement of other tissues [12, 62].

Our data demonstrate that PrP^C^ expression is widespread in white-tailed deer and confirms that symptomatic, CWD-positive, white-tailed deer have widespread prion seeding activity. Several questions arise from this work: 1) What is the role of local, cellular PrP^C^ in the generation of prions that are excreted by infected cervids? CWD prions have been detected in a number of excreta from cervids [30, 41, 42, 63–66] and in the organs that generate those excreta [30], but we are left to wonder whether the misfolding event that produced those prions occurred in the excretory organ, or whether the prions were transported there. 2) Can many organs be used for CWD diagnosis in deer? Our data suggest that RT-QuIC may permit the testing of a broad array of tissues and that many tissues have detectable prion seeding activity later in disease progression. 3) What mechanism explains the presence of prion seeding activity in alimentary tissues before neuroinvasion? Our data suggest that prions replicate in tissues of the alimentary tract before they reach the brain. This work contributes to the growing body of evidence that CWD prions are widespread within cervids and that PrP^C^ expression alone does not dictate the kinetics of prion spread in the body.

## Supporting information

S1 Table Description of PrP^C^ expression patterns. We described the PrP^C^ distribution in the alimentary and alimentary-associated lymphoid tissues investigated in this manuscript. This table contains the histologic descriptions of the staining patterns for each tissue. Patterns were conserved among deer and every tissue type was compared to a specimen stained with an isotype-control antibody, which were consistently negative.
(DOCX)
