# Supplementary material for: PrPC expression and prion seeding activity in the alimentary tract and lymphoid tissue of deer
Source: PLoS One. 2017 Sep 7;12(9):e0183927. doi: 10.1371/journal.pone.0183927 (PMC5589181; doi:10.1371/journal.pone.0183927)
Supplement: S1 Table — We described the PrPC distribution in the alimentary and alimentary-associated lymphoid tissues investigated in this manuscript. This table contains the histologic descriptions of the staining patterns for each tissue. Patterns were conserved among deer and every tissue type was compared to a specimen stained with an isotype-control antibody, which were consistently negative. (DOCX) [file pone.0183927.s001.docx]

| **Tissue** | **PrP^C^ pattern** |
| --- | --- |
| Alimentary system | |
| Rumen | Marked granular cytoplasmic staining diffusely in stratum granulosum and spinosum of mucosal stratified squamous epithelium; lesser staining in basal epithelium. No staining of superficial keratin. |
| Reticulum | Similar to rumen. Marked granular cytoplasmic staining diffusely in stratum granulosum, similar pattern but lesser staining in stratum spinosum, of mucosal stratified squamous epithelium. No staining of superficial keratin. |
| Omasum | Epithelial cells in stratum granulosum display diffuse cytoplasmic staining. |
| Abomasum | Individual cells in the gastric pits consistent with peptic chief cells display cytoplasmic staining. Percentage of stained cells varied with anti-PrP^C^ antibody. |
| Duodenum | Diffuse staining of crypt and villar enterocytes with varying degrees of intensity. No immunoreactivity observed in Bruner’s glands. |
| Jejunum | Diffuse staining of crypt and villar enterocytes with varying degrees of intensity. In Peyer’s patches, diffuse staining in germinal center of lymphoid follicle and marked cytoplasmic staining in individual histiocytic-like cells. Cytoplasmic staining in follicular-associated epithelium overlying Peyer’s patches. Diffuse immunoreactivity in myenteric plexi. |
| Ileum | In Peyer’s patches, diffuse staining in germinal centers of lymphoid follicles. Cytoplasmic staining in individual histiocytic-like cells and occasionally staining in lymphocytes. Marked diffuse staining of myenteric plexi. |
| Cecum | Marked staining of follicular germinal centers and myenteric plexi. Cytoplasmic staining of individual histiocytic-like cells in the mucosal lamina propria. |
| Colon | A mild number of individual histiocytic cells in the mucosal lamina propria displayed cytoplasmic staining. Marked staining in myenteric plexi. |
| Rectum | No staining in mucosa. Intense staining in myenteric plexi. Marked staining of follicle germinal centers. Mild number of histiocytic cells in the mucosa and submucosa. |
| Lymphoid tissues | |
| Tonsil | Cytoplasmic staining of surface mucosal epithelial cells. Diffuse staining in germinal centers of lymphoid follicles that extends to the mantle zone when present. Occasional cytoplasmic staining of histiocytic-like cells in paracortex and interstitial connective tissues. |
| Retropharyngeal LN | Diffuse staining in germinal centers which occasionally extends to the paracortex. Mild numbers of histiocytic-like cells in the subcapsular sinus and medullary sinus display cytoplasmic staining. |
| Mandibular LN | Diffuse staining in germinal centers of lymphoid follicles that occasionally extends into the mantle zone. A mild number of individual histiocytic-like cells in the medulla and paracortex display cytoplasmic staining. |
| Parotid LN | Diffuse staining in germinal centers of lymphoid follicles that occasionally extends into the mantle zone. |
| Mesenteric LN | Diffuse staining in germinal centers of lymphoid follicles and occasional staining of the paracortex. Mild number of histiocytic-like cells display cytoplasmic staining. Marked staining of peripheral nerves in adjacent mesentery. |
| Ileocecocolic LN | Diffuse staining in germinal centers of lymphoid follicles that extends to the mantle zone when present. |
| Prescapular LN | Diffuse staining in germinal centers of lymphoid follicles. Multifocal regional staining of cells in paracortex. |
| RAMALT | Diffuse intense staining of the rectal myenteric plexi. Marked staining of germinal centers. |
| Spleen | Diffuse staining in germinal centers that occasionally extends to mantle zone when present. Marked staining of peripheral nerves in trabeculae. Moderate number of histiocytic-like cells in the sinuses have cytoplasmic staining. |
| Nervous system | |
| Obex | Diffuse neuropil staining with greater intensity of grey matter compared with white matter. |
| Secretory tissues | |
| Parotid salivary gland | Diffuse staining in acinar cells. |
